# Comprehensive Bioinformatics Analysis to Identify the Gene HMMR Associated With Lung Adenocarcinoma Prognosis and Its Mechanism of Action in Multiple Cancers

**DOI:** 10.3389/fonc.2021.712795

**Published:** 2021-10-06

**Authors:** Jianguang Shi, Yingqi Chen, Zishan Wang, Jin Guo, Changyong Tong, Jingjie Tong, Wentao Hu, Chenwei Li, Xinjian Li

**Affiliations:** Thoracic Surgery Department, Ningbo First Hospital, Ningbo, China

**Keywords:** LUAD, DEGs, HMMR, overall survival, pan-cancer, cell cycle

## Abstract

**Background:**

Lung cancer is the third most frequently diagnosed cancer in the world, with lung adenocarcinoma (LUAD) as the most common pathological type. But studies on the predictive effect of a single gene on LUAD are limited. We aimed to discover new predictive markers for LUAD.

**Methods:**

Differentially high-expressed genes at each stage were obtained from the TCGA and GTEx databases. The functions of these genes were investigated through GO enrichment and KEGG pathway analyses. Then, the key genes were selected by applying whole gene overall survival time. The expression of the key gene was studied in LUAD, and survival analysis was performed using Kaplan-Meier mapper, followed by univariate and multifactorial COX analysis. Finally, the gene expression and its prognostic significance in the pan-cancer were examined.

**Results:**

A total of 10,106 DEGs were obtained from the two datasets. The top 266 differentially upregulated genes intersected with the top 1,497 overall survival-related genes, and 87 key genes were identified. High-expressed HMMR was associated with a poor prognosis of LUAD. Univariate and multifactorial Cox analysis showed that HMMR was an independent prognostic factor for LUAD patients. A high HMMR expression was strongly associated with the overall survival (OS) and disease-specific survival (DSS) in 11 cancer types and with poorer OS, DSS, and PFI in 10 cancer types.

**Conclusion:**

HMMR may be an independent prognostic indicator and an important biomarker in diagnosing and predicting the survival of LUAD patients. Also, HMMR may be a key predictor of a variety of cancers.

## Introduction

Lung cancer is a leading cause of cancer death worldwide without effective treatment (1). Lung adenocarcinoma (LUAD) is a subtype of lung cancer with high incidence and mortality rate, accounting for approximately more than half of all lung cancer cases (2). Compared with other subtypes, LUAD progresses more slowly and also has a greater chance of being detected after metastasis (3). If detected early, the survival of patients will be greatly improved. It has been pointed out that many biomarkers are closely related to tumor development and can be used as prognostic and predictive biomarkers of tumors (4, 5), indicating the significance of screening more molecular biomarkers.

In the recent years, an increasing number of prognostic biomarkers for LUAD have been identified through analyzing the clinical information and gene expression profiles of patients stored in public databases. For example, Wei et al. (6) analyzed the TCGA dataset expression profiles and screened 151 key genes associated with a relapse-free survival (RFS). After analyzing nine key genes with PPI, four out of nine genes were considered as prognostic biomarkers for patients with stage I LUAD. Some differentially expressed genes, such as CCR2, PTPRC, KIF4A, CCNB1, BUB1B, CDC20, TTK, MAD2L1, UBB, RAC1, and ITGB1, have also been regarded as possible biomarkers for LUAD prognosis and treatment (7–10). However, the validity of these diagnostic models in the clinical practice has not been tested, and the pathogenesis of LUAD is still unclear. Investigators have identified several potential biomarkers which could provide a clinical basis for the diagnosis, treatment, and prognosis of patients with LUAD. But still, effective prognostic markers and accurate therapeutic targets is still an urgent demand for patients with LUAD. Therefore, in this study, a bioinformatics approach was applied to screen the target genes that are closely related to the prognosis of LUAD patients, and to find prognostic biomarkers for LUAD.

In this study, we studied DEGs in LUAD using the Limma package of R software. Then, by applying one-way COX analysis to screen genes associated with the overall survival of LUAD, DEGs and their overlapping genes were screened to identify key genes. The expression of the key gene in LUAD and its relationship with survival prognosis were investigated. Finally, the role of the gene in multiple tumors was observed. We aimed to identify the biomarkers that are closely associated with the prognosis of LUAD and to analyze their prognostic predictive significance in LUAD and pan-cancer.

## Materials and Methods

### Data Collection

The gene expression data and clinical information from normal and tumor tissues involving 33 cancer types were downloaded from The Cancer Genome Atlas (TCGA)database (https://portal.gdc.cancer.gov/) and Genotype-Tissue Expression Project(GTEx)database (https://gtexportal.org/). Tissue cell line analysis of HMMR was performed using the CCLE database (https://portals.broadinstitute.org/). The gene expression matrix and clinical information of 513 LUAD tumor samples were downloaded from the TCGA database (https://portal.gdc.cancer.gov/). Abbreviations of the tumor name and their corresponding meanings are given in **Table 1**. Different HMMR expression levels in tumor tissues and normal tissues were analyzed by the edgeR software. The Kruskal-Wallis test was used to determine the expression of HMMR in different normal tissues and different tumor cell lines. Violin plots were drawn by the R package ggplot.

**Table 1:** Tumor name abbreviations and their corresponding meanings.

Abbreviations	Tumor name
ACC	Adrenocortical Carcinoma
BLCA	Bladder Urothelial Carcinoma
BRCA	Breast invasive carcinoma
CESC	Cervical squamous cell carcinoma and endocervical adenocarcinoma
CHOL	Cholangiocarcinoma
COAD	Colon adenocarcinoma
DLBC	Lymphoid Neoplasm Diffuse Large B-cell Lymphoma
ESCA	Esophageal carcinoma
GBM	Glioblastoma multiforme
HNSC	Head and Neck squamous cell carcinoma
KICH	Kidney Chromophobe
KIRC	Kidney renal clear cell carcinoma
KIRP	Kidney renal papillary cell carcinoma
LAML	Acute Myeloid Leukemia
LGG	Brain Lower Grade Glioma
LIHC	Liver hepatocellular carcinoma
LUAD	Lung adenocarcinoma
LUSC	Lung squamous cell carcinoma
MESO	Mesothelioma
OV	Ovarian serous cystadenocarcinoma
PAAD	Pancreatic adenocarcinoma
PCPG	Pheochromocytoma and Paraganglioma
PRAD	Prostate adenocarcinoma
READ	Rectum adenocarcinoma
SARC	Sarcoma
SKCM	Skin Cutaneous Melanoma
STAD	Stomach adenocarcinoma
TGCT	Testicular Germ Cell Tumors
THCA	Thyroid carcinoma
THYM	Thymoma
UCEC	Uterine Corpus Endometrial Carcinoma
UCS	Uterine Carcinosarcoma
UVM	Uveal Melanoma

### Differentially Expressed Gene Identification

All data were carefully studied for differential expression of mRNA using the Limma package of R software (version: 3.40.2).”P-value < 0.05 and |log2(FC)| > 1” were defined as the threshold of mRNA differentially expressed genes (DEGs).

### Gene Ontology and Kyoto Encyclopedia of Genes and Genomes Enrichment Analysis

GO analysis and KEGG analysis were performed on the common DEGs for each data using the DAVID 6.8 database (https://david.ncifcrf.gov/). Enrichment results with p < 0.05 or FDR < 0.05 were defined as statistically significant.

### Key Gene Screening

After DEGs analysis and Kaplan-Meier analysis, several significantly expressed genes were obtained and selected for potential key genes by “VennDiagram” in the R package.

### Kaplan-Meier Analysis

The survival information of all samples was obtained from the TGCA database to further analyze the relationship between the gene expression and survival rate. For Kaplan-Meier curves, p-values and hazard ratios (HR) with 95% confidence intervals (CI) were derived by performing log-rank test and univariate Cox proportional hazards regression. All of the above analytical methods and R packages were conducted using the v4.0.3 version of the R software (R Foundation for Statistical Computing, 2020). A p < 0.05 was considered as statistically significant.

### COX Regression Model

Univariate and multivariate cox regression analyses and forest plots were performed using the “forest plot” R package to show the P-values, HRs, and 95% CIs for each variable. Based on the results of multivariate Cox proportional risk analysis, column line plots were created using the R package “RMS” to predict the overall recurrence rate at 1, 3, and 5 year(s). The line graphs provide a graphical representation of these factors and allow the prognostic risk of an individual patient to be calculated from the points associated with each risk factor.

### Immunological Correlation Analysis

Data of the scores of six immune infiltrating cells for 33 cancers were downloaded from the TIMER database, and the correlation of gene expression with these immune cell scores was separately analyzed. The immune scores and stromal scores of individual tumor samples were studied using the R package ESTIMATE to analyze gene expression against immune scores in 33 tumors.

### Gene Set Enrichment Analysis

To explore the effect of gene expression on tumors, the samples were divided into two groups of high and low expression according to the gene expression. Enrichment of the KEGG and HALLMARK pathways in the high- and low-expression groups was analyzed using the GSEA tool downloaded from the Broad Institute (http://software.broadinstitute.org/gsea/downloads.jsp). KEGG is a comprehensive database incorporating genomic, chemical, and systematic functional information. Moreover, for GSEA analysis, the molecular signatures database (MsigDB, http://software.broadinstitute.org/gsea/msigdb) with the Hallmark gene set was also employed. Pathways were considered significantly enriched when they met the sub-conditions of |NES| > 1, p-value < 0.05, FDR < 0.25 (the threshold of GSEA).

## Results

### Results of Differentially Expressed Gene Screening in Lung Adenocarcinoma

**Figure 1:**
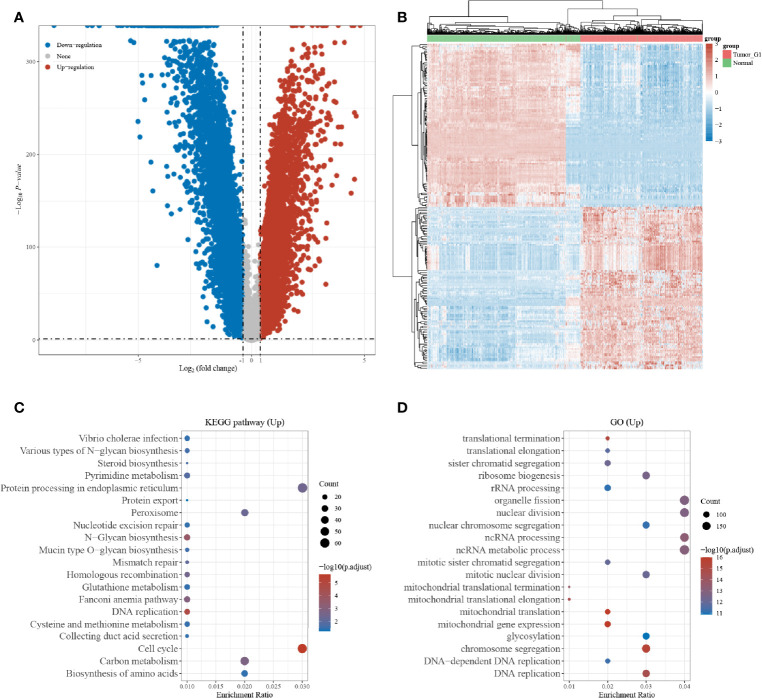
Differentially expressed gene screening; **(A)** Volcano map of differentially expressed genes in the TCGA and GTEx datasets; **(B)** Heat map of differentially expressed genes in the TCGA and GTEx datasets; **(C)** KEGG enrichment analysis of differentially expressed genes; **(D)** GO enrichment analysis of differentially expressed genes.

A total of 10,106 differentially expressed genes, including 4,800 upregulated genes and 5,306 downregulated genes, were obtained according to the Limma package analysis of the R software. The volcano plot (**Figure 1A**) and heat map **(** **Figure 1B**) were plotted using the Fold change and corrected p-value values. KEGG pathway analysis was then performed on the differentially upregulated genes, and a total of 20 pathways were found to be involved in the differentially upregulated genes, and were mainly enriched to the endoplasmic reticulum protein processing signaling pathway, and cell cycle signaling pathway that showed the highest enrichment (**Figure 1C**). GO enrichment analysis demonstrated that the differentially upregulated genes were mainly enriched to organelle division, nuclear division, non-coding RNA processing, and non-coding RNA metabolism (**Figure 1D**).

### Whole-Gene Survival Analysis and Overlap Screening of Differentially Upregulated Genes in Lung Adenocarcinoma

**Figure 2:**
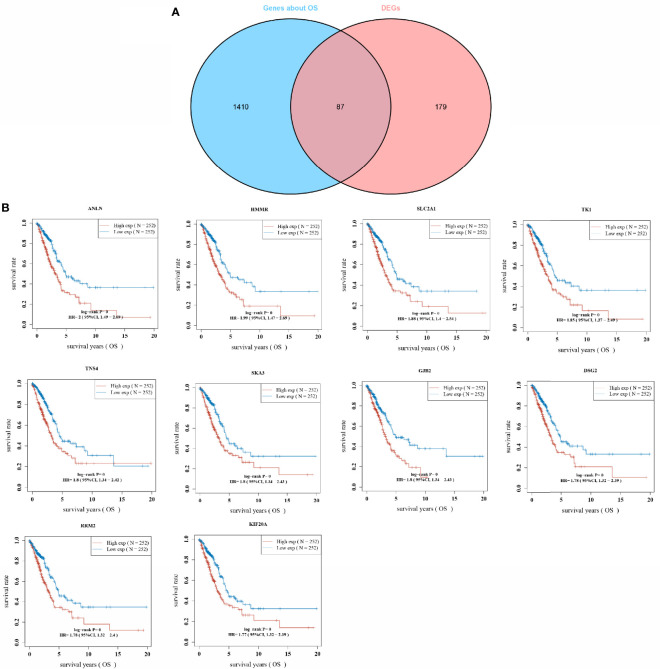
Overlapping gene screening; **(A)** Wayne diagram showing genes in tumors with one-way cox analysis of significant genes *versus* overlapping genes in DEGs; **(B)** KM curves for the top 10 most significantly different genes in overlapping genes.

To better determine the key genes, the relationship between the expression of whole genes in LUAD and the overall survival was analyzed with one-way COX risk analysis. The top 1,497 genes that were significantly associated with survival were selected, and 87 key genes were identified after overlapping gene analysis on the top 267 differentially upregulated genes (**Figure 2A**). The expression of the 87 key genes was further analyzed for their effect on the survival prognosis of LUAD. **Figure 2B** shows the KM curves of the top 10 genes in LUAD. The expression of ANLN gene expression in LUAD has been studied by scholars (11), while the relationship between HMMR overexpression and LUAD and pan-cancer prognosis has been less investigated, which was therefore studied in the present research.

### Analysis of Hyaluronan-Mediated Motility Factor Receptor Expression and Lung Adenocarcinoma Survival

**Figure 3:**
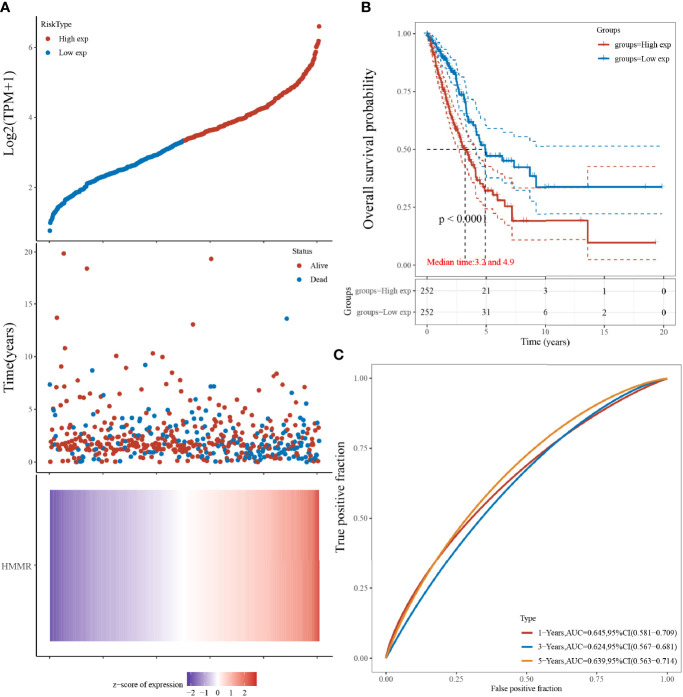
HMMR expression and LUAD survival analysis; **(A)** Gene expression *versus* survival time and survival status; **(B)** KM survival curve distribution of HMMR expression in the TCGA dataset; **(C)** ROC curve of HMMR at different times.

The results indicated that LUAD prognosis was significantly related to HMMR, and that a higher expression was predictive of a worse prognosis. The samples were sorted by gene expression from low to high, and divided into high-expression group (red) and low-expression group (blue). The survival time of patients with a high HMMR expression had a significantly higher mortality than those with a low HMMR. The HMMR expression heat map showed that HMMR was a risk factor (**Figure 3A**). From the KM survival curve (**Figure 3B**), patients with a high HMMR expression were associated with a poor prognosis. The ROC curve demonstrated that the 1-year, 2-year, and 3-year survival AUC were 0.680, 0.718, and 0.772, respectively, indicating that the model has a high accuracy.

### Hyaluronan-Mediated Motility Factor Receptor Expression and Prognostic Prediction Model of Lung Adenocarcinoma

**Figure 4:**
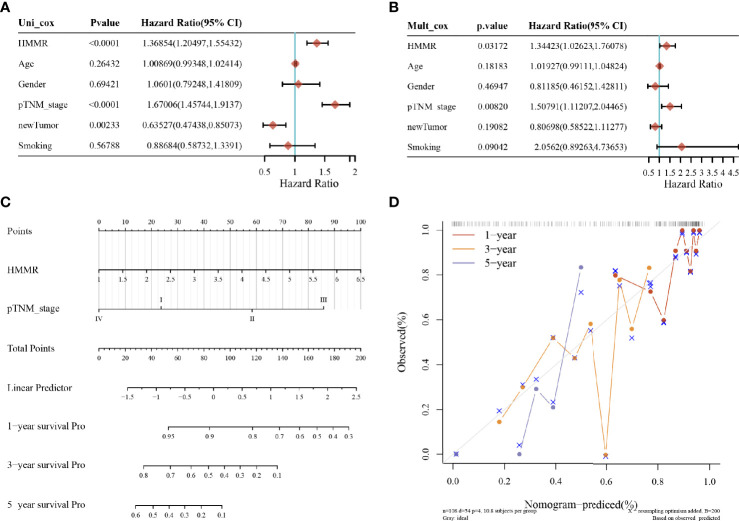
LUAD prognostic analysis; **(A)** value, risk factor HR, and confidence interval for single-factor cox analysis of gene expression and clinical characteristics; **(B)** value, risk factor HR, and confidence interval for multi-factor cox analysis of gene expression and clinical characteristics; **(C)** column line plot of HMMR *versus* other prognostic factors; **(D)** calibration curve of the column line plot.

The results of univariate and multivariate COX analyses showed that HMMR and TNM staging could be used as independent predictors for LUAD patients (**Figures 4A, B**). Subsequently, for validation, we constructed a prognostic analysis column line table for HMMR and TNM (**Figure 4C**), with higher scores on the column line table representing a worse prognosis. In the calibration curve (**Figure 4D**), the close 1, 3, and 5-year survival Nomogram model to the calibration curve indicated that the model could predict better results.

### The Relationship Between Hyaluronan-Mediated Motility Factor Receptor Expression and Immune Infiltration

**Figure 5:**
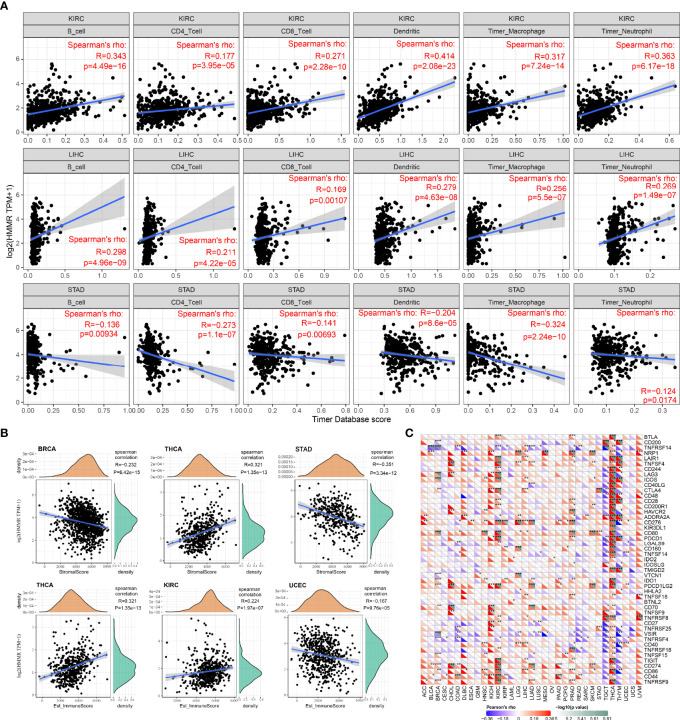
Relationship between HMMR expression and immune infiltration and immune cell subpopulations; **(A)** top three tumor types with a significant correlation between HMMR expression and immune infiltration; **(B)** top three tumor types with a significant correlation between HMMR expression and immune score and stromal score; **(C)** correlation between HMMR expression and immune-stimulating factors. *P < 0.05; **P < 0.01; ***P < 0.001.

Tumor-infiltrating lymphocytes are independent predictors of cancer precursor lymph node status and survival, therefore, the correlation between HMMR expression and immune infiltration levels in different cancer types was analyzed by the TIMER database. **Figure 5A** shows the top 3 tumor types from 33 cancer types screened according to the correlation between HMMR expression and the level of each immune cell infiltration. It was observed that in these three tumors, HMMR could influence the progression of cancer by regulating the immune cell infiltration. **Figure 5B** presents the top 3 tumor types in an immune score and stromal score of HMMR in 33 tumor types. The results showed that HMMR expression may affect tumorigenic progression by altering the tumor microenvironment in BRCA, THCA, STAD, HIRC, and UGEC. To verify the relationship between HMMR expression and immune function, we collected more than 40 common immune checkpoint genes, and analyzed the relationship between HMMR expression and immune checkpoint gene expression. As shown in **Figure 5C**, HMMR expression was positively correlated with the immune checkpoint genes in some tumors, particularly in KICH, KIRC, and THCA.

### Expression and Prognostic Analysis of Hyaluronan-Mediated Motility Factor Receptor in Various Tumors

**Figure 6:**
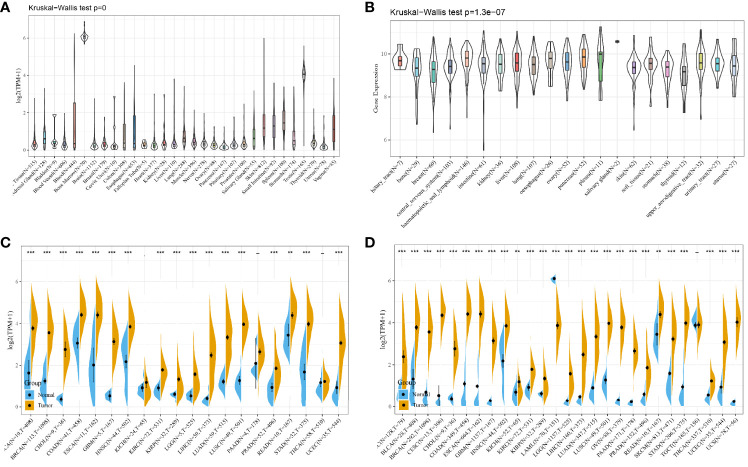
HMMR expression in pan-cancer; **(A)** HMMR expression level in each normal sample from the GTEx database source; **(B)** HMMR expression level in each tumor cell line from the CCLE database source; **(C)** HMMR expression level in each tumor sample from the TCGA database source; **(D)** HMMR expression level in samples from the TCGA and GTEx database sources. **P < 0.01; ***P < 0.001.

From the results of the GTEx database analysis, it could be found that the expression of HMMR in different normal tissues was inconsistent (**Figure 6A**). The results of the CCLC database analysis showed that the expression of HMMR in different tumor cell lines was also inconsistent (**Figure 6B**). Because the number of normal samples in the TCGA database was small (**Figure 6C**), further analysis of HMMR expression in 27 tumors was performed by integrating data from normal tissues in the GTEx database and TCGA tumor tissues (**Figure 6D**). Here, the results did not present statistically significant differences in HMMR expression in testicular cancer (TGCT). Low expression of HMMR in acute myeloid leukemia (LAML) and its high expression in the remaining tumors was shown in the figure.

### Prognostic Analysis of Hyaluronan-Mediated Motility Factor Receptor in Pan-Cancer

**Figure 7:**
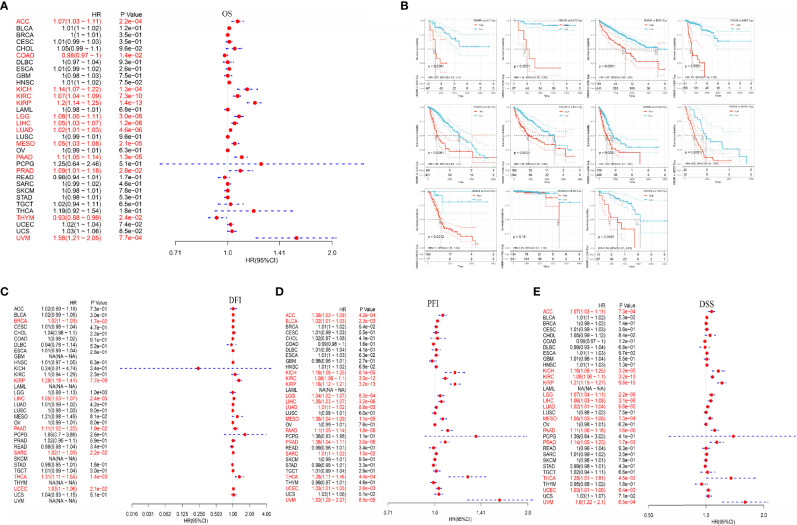
Analysis of HMMR expression and pan-cancer prognosis; **(A)** HMMR expression in different tumors correlated with the OS; **(B)** KM survival curve of high-risk genes; **(C)** HMMR expression in different tumors correlated with DFI; **(D)** HMMR expression in different tumors correlated with PFI; **(E)** HMMR expression in different tumors correlated with DSS.

After analyzing the expression profile data of 33 oncogenes in TCGA, the relationship between the gene expression and survival prognosis was studied using univariate Cox proportional risk regression model. The relationship between HMMR expression and the overall survival (OS) is shown in **Figure 7A**, and the results indicated that HMMR was associated with a poor prognosis of ACC, KICH, KIRC, KIRP, LGG, LIHC, LUAD, MESO, PAAD, PRAD, and UVM. The KM curves of high-risk genes are shown in **Figure 7B**. The correlation between HMMR expression and DFI, PFI, and DSS correlation is shown in **Figures 7C–E**.

### Gene Set Enrichment Analysis

**Figure 8:**
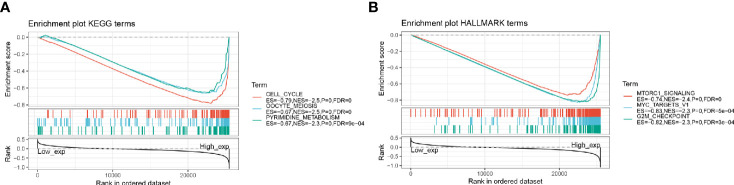
GSEA enrichment analysis; **(A)** HMMR high expression in KEGG significantly enriched pathway; **(B)** HMMR high expression in HALLMARK significantly enriched pathway.

The biological characteristics of HMMR in LUAD were analyzed by GSEA enrichment. The GSEA results showed that a high expression of HMMR in the KEGG-related pathway was mainly enriched to the cell cycle, oocyte meiosis, and pyrimidine metabolism (**Figure 8A**). HALLMARK-related pathway was mainly enriched to the MTORC1 signaling system, MYC target, and G2M checkpoint (**Figure 8B**).

## Discussion

Hyaluronan-mediated motility factor receptor (HMMR), also known as RHAMM/CD168, is a protein with multiple cellular functions. It was initially identified as a soluble substance used to bind hyaluronic acid, a component of the extracellular matrix, which is a product secreted by fibroblasts. HMMR mainly plays a role in response to tissue injury and wound repair (12, 13). A study also showed that HMMR could promote cell cycle progression by participating in microtubule spindle formation and activate signaling pathways that enhances cell migration (14). In this study, a total of 10,106 differentially expressed genes were obtained after the expression profiling of LUAD samples, and HMMR was among the differentially upregulated genes. This indicated that HMMR expression was upregulated in LUAD. Hyaluronic acid (HA) can be preferentially detected in high-grade lung cancer mesenchyme (15), and hyaluronic acid-mediated motor factor receptor (HMMR) expression was correlated with the prognostic survival of LUAD patients (16). The present study also confirmed that HMMR could be an independent prognostic factor for LUAD patients with univariate and multifactorial COX analysis. The work of Xiao et al. (17) identified key differentially expressed genes associated with non-small cell lung cancer by bioinformatics analyses. He et al. (18) established a robust 8-gene prognostic signature for early-stage non-small cell lung cancer, and the signature also includes HMMR. These previous reports, together with our current, results indicated that HMMR may play important roles in LUAD.

By integrating normal tissue data from the GTEx database and TCGA tumor tissue data, further analysis of HMMR expression in 27 tumors showed that HMMR was high-expressed in the majority of tumors. This result was the same as previously reported studies (19). In addition, we explored the relationship between HMMR expression and the prognosis of multiple tumors, and found that a high HMMR expression was a protective factor in thymic carcinoma and colon adenocarcinoma, with a higher expression indicating a better prognosis. An increasing number of reports suggested that the tumor immune microenvironment plays an important role in tumor development. Combining both the immune score and stromal score, our analysis revealed that HMMR influenced tumor development through the tumor microenvironment in KIRC. Previous studies have shown that the short peptide HMMR can be effectively presented by DC cells, activate T cells, and induce immune responses (7, 20, 21). HMMR-T cell therapy engineered with T cell receptor (TCR-T) could be able to effectively inhibit the tumor growth in animal models (22). In the recent years, with the breakthrough of immune checkpoints, ICIs have been increasingly used in the field of tumor immunotherapy and obtained some encouraging results. MacKay et al. (23) suggested that HMMR could be a potential target for chimeric antigen receptor T-cell immunotherapy (CAR-T). Therefore, HMMR immune agents have great potentials in research.

Finally, we used GSEA enrichment to analyze the biological functions of HMMR in tumors, and the results showed that HMMR may be involved in biological functions such as cell cycle, oocyte meiosis, and pyrimidine metabolism. It has also been previously shown that HMMR expression is regulated by the cell cycle, with peak expression between late G2 and early mitosis (24). The latest research also demonstrated that HMMR is a glycolysis-related gene as a potential prognostic marker in non-small cell lung cancer (25). HMMR is also an aberrantly expressed gene associated with the cell cycle in proliferating cells of patients with acute myeloid leukemia *in vitro* (26). HMMR regulates the spindle assembly during mitosis and meiosis (27). In mouse models, mutations or deletions in HMMR/HMMR expression can also disrupt the proper development or homeostasis of gonadal tissues (26, 28). Although the mechanism is unclear, this may indicate the ability of a high HMMR expression in the gonads to affect gamete formation through regulating meiosis (29).

Although the functional role of HMMR in LUAD was computationally explored and analyzed, and the results are of clinical significance, some limitations should be acknowledged. Bioinformatics methods used for gene identification lacks novelty to identify the prognostic signature, more grouped variable selection methods should be used. Experiments were not conducted, which is another limitation in this study. In the future, more experiments and large-scale clinical trials are needed to further validate these findings.

## Conclusion

In this study, we identified the gene HMMR as an independent prognostic biomarker for lung adenocarcinoma (LUAD) based on an integrative bioinformatics analysis. The result indicated that a high expression of HMMR was associated with a poor prognosis of LUAD. Univariate and multifactorial Cox analysis further confirmed the prognostic significance of HMMR. In addition, HMMR was found to be functional in a variety of cancers. These findings may contribute to the clinical decision-making of an individualized treatment for LUAD patients.

## Data Availability Statement

The original contributions presented in the study are included in the article/supplementary material. Further inquiries can be directed to the corresponding authors.

## Author Contributions

All authors contributed to the article and approved the submitted version.

## Funding

This work was supported by the Ningbo Natural Science Foundation Project (2018A610274) The expression and biological function of microRNA miR-542-3p in NSCLC patients” under grant NO.2018A610274 and the Ningbo Medical Association Foundation Project (2017A46) “The clinical significance of circular RNA CDR1as in the development of non-small cell lung cancer” under grant NO.2017A46.

## Conflict of Interest

The authors declare that the research was conducted in the absence of any commercial or financial relationships that could be construed as a potential conflict of interest.

## Publisher’s Note

All claims expressed in this article are solely those of the authors and do not necessarily represent those of their affiliated organizations, or those of the publisher, the editors and the reviewers. Any product that may be evaluated in this article, or claim that may be made by its manufacturer, is not guaranteed or endorsed by the publisher.
